# Supplementary material for: The amygdala instructs insular feedback for affective learning
Source: eLife. 2020 Nov 20;9:e60336. doi: 10.7554/eLife.60336 (PMC7679142; doi:10.7554/eLife.60336)
Supplement: Transparent reporting form [file elife-60336-transrepform.docx]

***eLife’s* transparent reporting form**

We encourage authors to provide detailed information *within their submission* to facilitate the interpretation and replication of experiments. Authors can upload supporting documentation to indicate the use of appropriate reporting guidelines for health-related research (see [EQUATOR Network](http://www.equator-network.org/%20)), life science research (see the [BioSharing Information Resource](https://biosharing.org/)), or the [ARRIVE guidelines](http://www.plosbiology.org/article/info:doi/10.1371/journal.pbio.1000412) for reporting work involving animal research. Where applicable, authors should refer to any relevant reporting standards documents in this form.

If you have any questions, please consult our Journal Policies and/or contact us: [editorial@elifesciences.org](mailto:editorial@elifesciences.org).

**Sample-size estimation**

- You should state whether an appropriate sample size was computed when the study was being designed
- You should state the statistical method of sample size computation and any required assumptions
- If no explicit power analysis was used, you should describe how you decided what sample (replicate) size (number) to use

Please outline where this information can be found within the submission (e.g., sections or figure legends), or explain why this information doesn’t apply to your submission:

Sample size are taken from previous literature and found to be in line with sample size estimates calculated from data in our lab. This is described in the ‘Data analyses and statistical tests’ section in Materials and Methods.

**Replicates**

- You should report how often each experiment was performed
- You should include a definition of biological versus technical replication
- The data obtained should be provided and sufficient information should be provided to indicate the number of independent biological and/or technical replicates
- If you encountered any outliers, you should describe how these were handled
- Criteria for exclusion/inclusion of data should be clearly stated
- High-throughput sequence data should be uploaded before submission, with a private link for reviewers provided (these are available from both GEO and ArrayExpress)

Please outline where this information can be found within the submission (e.g., sections or figure legends), or explain why this information doesn’t apply to your submission:

Establishment of the behavioral assay, neural recordings, and circuit manipulation were performed in independent experiments with separate animal cohorts (Figure 2, 5, Figure 2—figure supplement 5, 7, Figure 5—figure supplement 3; biological replicates). Basic behavior was replicated across experiments for control groups. Circuit manipulations were replicated using different technologies on separate experiments and cohorts (Figure 2, Figure 2—figure supplement 5, 7; biological replicates). Neural activity recordings were replicated in independent animals (biological replicates) and across sessions within animals (technical replicates) (Figure 1, 5, Figure 1—figure supplement 5, 6, 8, Figure 5—figure supplement 5). For behavioral experiments, 8/97 animals were excluded for failing port training, low virus expression, or misplaced/broken fibers. For in vivo electrophysiology and calcium imaging, 4/14 and 13/26 animals were excluded due to absent Calcium signals or absent/low quality signals, respectively. After unit identification, no further animals were excluded in either case. The statement in this regard can be found in the Materials and Methods section ‘Data analyses and statistical tests’.

**Statistical reporting**

- Statistical analysis methods should be described and justified
- Raw data should be presented in figures whenever informative to do so (typically when N per group is less than 10)
- For each experiment, you should identify the statistical tests used, exact values of N, definitions of center, methods of multiple test correction, and dispersion and precision measures (e.g., mean, median, SD, SEM, confidence intervals; and, for the major substantive results, a measure of effect size (e.g., Pearson's r, Cohen's d)
- Report exact p-values wherever possible alongside the summary statistics and 95% confidence intervals. These should be reported for all key questions and not only when the p-value is less than 0.05.

Please outline where this information can be found within the submission (e.g., sections or figure legends), or explain why this information doesn’t apply to your submission:

Statistical details are reported in the Materials and Methods section ‘Data analyses and statistical tests’, figure legends and in Appendix 1-table 1.

(For large datasets, or papers with a very large number of statistical tests, you may upload a single table file with tests, Ns, etc., with reference to sections in the manuscript.)

**Group allocation**

- Indicate how samples were allocated into experimental groups (in the case of clinical studies, please specify allocation to treatment method); if randomization was used, please also state if restricted randomization was applied
- Indicate if masking was used during group allocation, data collection and/or data analysis

Please outline where this information can be found within the submission (e.g., sections or figure legends), or explain why this information doesn’t apply to your submission:

Animals were assigned randomly to all experimental cohorts. The behavioral experimenter was blind to the treatment of respective groups, wherever possible. Behavioral and neural data analyses was carried out blinded and computationally, wherever applicable. These statements can be found in the Materials and Methods section ‘Data analyses and statistical tests’.

**Additional data files (“source data”)**

- We encourage you to upload relevant additional data files, such as numerical data that are represented as a graph in a figure, or as a summary table
- Where provided, these should be in the most useful format, and they can be uploaded as “Source data” files linked to a main figure or table
- Include model definition files including the full list of parameters used
- Include code used for data analysis (e.g., R, MatLab)
- Avoid stating that data files are “available upon request”

Please indicate the figures or tables for which source data files have been provided:

Numerical data presented in the graphs are provided as source data in

Figure 1-source data 1

Figure 1-source data 2

Figure 2-source data 1

Figure 3-source data 1

Figure 4-source data 1

Figure 4-source data 2

Figure 5-source data 1

Figure 5-source data 2
